# Optical coherence tomography shows neuroretinal thinning in myelopathy of adrenoleukodystrophy

**DOI:** 10.1007/s00415-019-09627-z

**Published:** 2019-11-12

**Authors:** Wouter J. C. van Ballegoij, Sander C. Kuijpers, Irene C. Huffnagel, Henry C. Weinstein, Bwee Tien Poll-The, Marc Engelen, Carlien A. M. Bennebroek, Frank D. Verbraak

**Affiliations:** 1 Department of Paediatric Neurology/Emma Children’s Hospital, Amsterdam UMC, University of Amsterdam, Meibergdreef 9, 1100DD Amsterdam, The Netherlands; 2 Department of Neurology, OLVG Hospital, Amsterdam, The Netherlands; 3 Department of Ophthalmology, Amsterdam UMC, University of Amsterdam, Amsterdam, The Netherlands

**Keywords:** X-linked adrenoleukodystrophy, Myelopathy, Optical coherence tomography, Neurodegeneration, Retinal nerve fiber layer, Spinal cord

## Abstract

**Background:**

Progressive myelopathy is the main cause of disability in adrenoleukodystrophy (ALD). Development of therapies is hampered by a lack of quantitative outcome measures. In this study, we investigated whether myelopathy in ALD is associated with retinal neurodegeneration on optical coherence tomography (OCT), which could serve as a surrogate outcome measure.

**Methods:**

Sixty-two patients (29 men and 33 women) and 70 age-matched and sex-matched controls (33 men and 37 women) were included in this cross-sectional study. We compared retinal nerve fiber layer (RNFL), ganglion cell layer (GCL) and peripapillary retinal nerve fiber layer (pRNFL) thickness between ALD patients and controls. In addition, we correlated these OCT measurements with clinical parameters of severity of myelopathy.

**Results:**

Patients had significantly thinner RNFL (male group, *p* < 0.05) and pRNFL superior and temporal quadrant [both male (*p* < 0.005) and female (*p* < 0.05) groups] compared to controls. Comparing three groups (symptomatic patients, asymptomatic patients and controls), there were significant differences in RNFL thickness (total grid and peripheral ring) in the male group (*p* ≤ 0.002) and in pRNFL thickness (superior and temporal quadrant) in both male (*p* ≤ 0.02) and the female (*p* ≤ 0.02) groups. Neuroretinal layer thickness correlated moderately with severity of myelopathy in men (correlation coefficients between 0.29–0.55, *p* < 0.02), but not in women.

**Conclusions:**

These results suggest that neurodegeneration of the spinal cord in ALD is reflected in the retina of patients with ALD. Therefore, OCT could be valuable as an outcome measure for the myelopathy of ALD. Additional longitudinal studies are ongoing.

**Electronic supplementary material:**

The online version of this article (10.1007/s00415-019-09627-z) contains supplementary material, which is available to authorized users.

## Introduction

Myelopathy is the main clinical manifestation and cause of disability in X-linked adrenoleukodystrophy (ALD, OMIM 300100) [1, 2]. ALD is a genetic neurometabolic disorder caused by a defect in the degradation of very long-chain fatty acids (VLCFA), leading to their accumulation in various tissues [3, 4]. Virtually all men with ALD develop myelopathy, characterized neuropathologically by degeneration of the corticospinal tracts, spinothalamic tracts and dorsal columns of the spinal cord [5, 6]. Clinically, it presents as a slowly progressive gait disorder due to a spastic paraparesis and sensory ataxia [7]. Despite the X-linked inheritance, over 80% of women with ALD (heterozygotes) also develop myelopathy, although at a later age and with slower progression than men [8, 9]. Treatment is currently supportive only, but new disease-modifying therapies are being developed [10]. For these therapies to be tested in clinical trials, there is a need for reliable and sensitive quantitative outcome measures.

Measuring the severity and progression of myelopathy in ALD, however, is problematic. Neurological examination and current clinical outcome measures are subject to a high intra- and interrater variability [11, 12]. Moreover, disease progression is very slow, occurring over years or even decades [13]. Our group recently showed that statistically significant progression of myelopathy in men with ALD can be measured during 2-year follow-up using clinical outcome measures, but absolute changes were small [14]. Clinical trials using these outcome measures require a long treatment period (at least 2 years) and a large number of patients to be able to detect differences between treatment arms. Therefore, more sensitive and reproducible surrogate outcome measures for myelopathy in ALD are needed.

Spectral domain optical coherence tomography (SD-OCT) is a rapid, noninvasive, safe and (provided that subjects are followed on the same scanner) reproducible technique to visualize the retina in vivo [15–17]. It provides cross-sectional images of the macula and optic nerve head with enough resolution to accurately measure thickness of the individual retinal layers. Degeneration of some of these layers, especially the retinal nerve fiber layer (RNFL, containing the axons of neurons projecting from the retina to the thalamus) and ganglion cell layer (GCL, containing the cell bodies of these neurons), is associated with disease severity and progression in neurodegenerative diseases such as Alzheimer and Parkinson’s disease [18, 19], but also with neuro-axonal degeneration in multiple sclerosis and amyotrophic lateral sclerosis [20–22]. These studies suggest that the neurodegeneration occurs simultaneously in the central nervous system and retina. As axonal degeneration is the pathological hallmark of myelopathy in ALD, thinning of RNFL and the GCL could reflect spinal cord damage and, therefore, serve as a surrogate outcome measure for myelopathy in ALD. Indeed, thinning of the RNFL has been reported in an ALD patient with myelopathy [23], but has never been systematically studied in a larger group of ALD patients. Therefore, in this cross-sectional study, we investigated the association between retinal neurodegeneration, measured as RNFL and GCL thickness on OCT, and the severity of myelopathy in both men and women with ALD. As myelopathy in women with ALD has a milder disease course than in men, we hypothesized that retinal neurodegeneration would be less pronounced in the female subgroup.

## Methods

### Study design and participants

This cross-sectional study was part of a large observational cohort study on the natural history of ALD (the Dutch ALD cohort). Patients were recruited at the Amsterdam UMC (Amsterdam, the Netherlands) between June 2015 and March 2018. Patients over 16 years of age with a confirmed diagnosis of ALD were eligible to participate. We excluded patients with active cerebral ALD (defined as gadolinium-enhancing white matter lesions on MRI), diabetes mellitus, a history of neurodegenerative or ophthalmological disease and any comorbidity interfering with the assessment of myelopathy.

Study participation for patients included one hospital visit with neurological assessment, ophthalmological examination, OCT imaging and MR imaging. The ophthalmological examination was performed by an experienced staff member and included visual acuity measurement (ETDRS card with Sloan letters), measurement of intraocular pressure with air-puff tonometry, slit-lamp biomicroscopy and fundus photography. We excluded eyes with low visual acuity (> 0.1 LogMar), high refractive errors (> 6 diopter), intra-ocular pressure > 21 mmHg, substantial media opacities and optic nerve disease or retinal disease as defined in the OSCAR-IB criteria [24]. MRI scans to exclude active cerebral ALD were evaluated by an experienced neuroradiologist. Sex- and age-matched controls without a history of diabetes, neurological or ophthalmological disease and a normal visual acuity (≤ 0.1 Logmar) were recruited via public advertisement.

### Neurological assessment

The protocol used to assess myelopathy in this cohort has been previously described [14, 25]. In short, patients underwent a detailed neurological history and examination. They were scored as symptomatic if they had both signs and symptoms of myelopathy. Clinical outcome measures used to quantify myelopathy were the Expanded Disability Status Score (EDSS), Severity Scoring system for Progressive Myelopathy (SSPROM) and timed up-and-go. The EDSS measures neurological disability ranging from 0 (no disability) to 10 (death) [26]. SSPROM measures severity of myelopathy ranging from 0 to 100, with lower scores indicating a higher degree of impairment [27, 28]. The timed up-and-go is used to assess walking function by recording the time that the patient needs to get up from an armchair, walk 3 meters, turn around, walk back and sit down again [29, 30]. Neurological assessments were done on the same day as OCT-imaging.

### Imaging protocol and image analysis

**Fig. 1:**
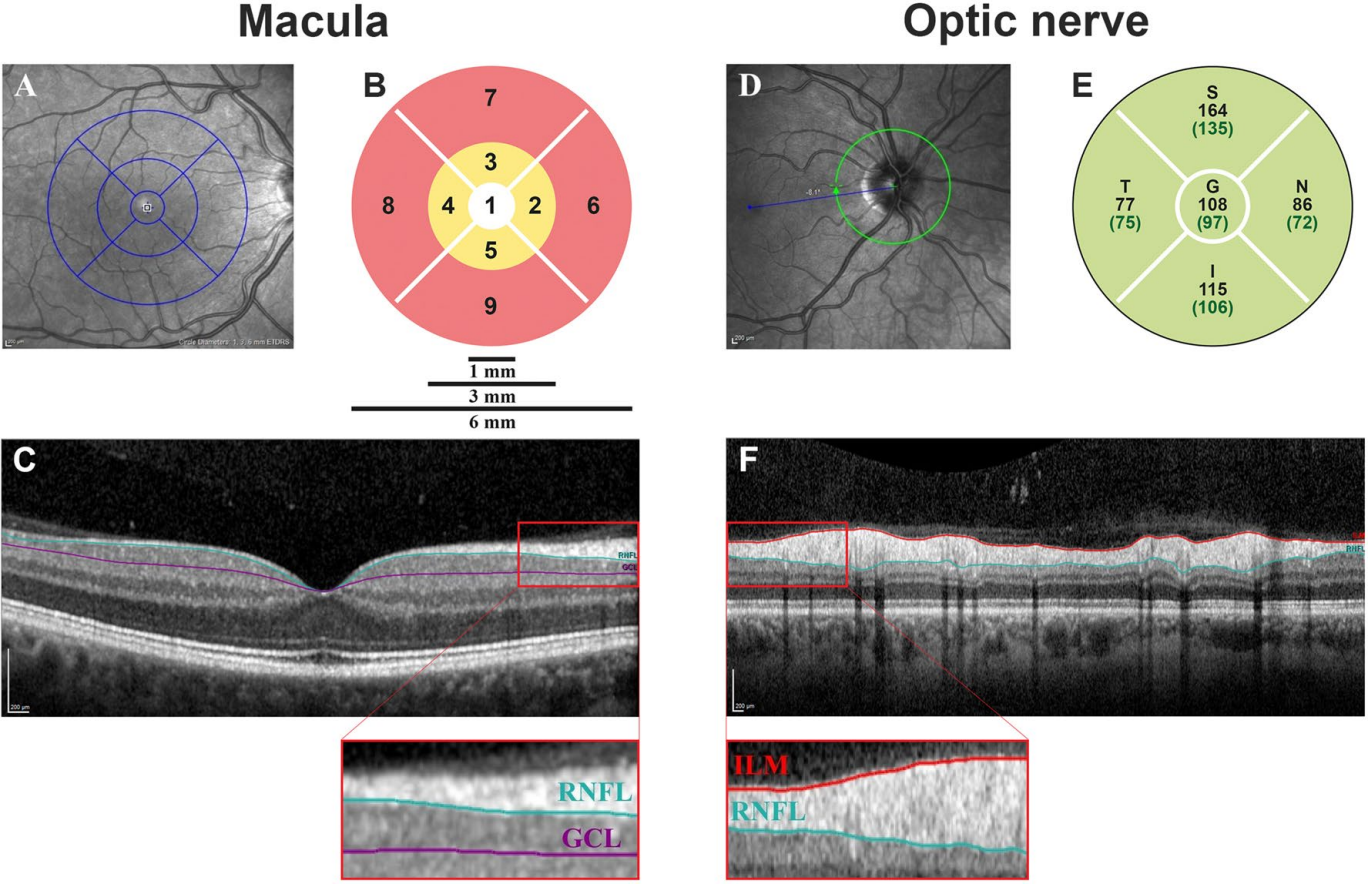
Optical coherence tomography output. The left panel shows a macular scan with **a** the Early Treatment Diabetic Retinopathy Study (EDTRS) grid **b** the pericentral (yellow) and peripheral (red) ring and **c** a cross-section of the retina showing the retinal nerve fiber layer (RNFL) and ganglion cell layer (GCL). The right panel shows an optic nerve scan with **d** the 3.5 mm peripapillary ring **e** the Heidelberg output of the peripapillary retinal nerve fiber layer (pRNFL) thickness and **f** a cross-section of the periparillary retina with the pRNFL

OCT-imaging was performed by three OCT-operators under dimmed-light conditions on two identical Heidelberg Spectralis OCT-scanners (Heidelberg Engineering GmbH, Germany). Images of both the macula and the optic nerve (peripapillary scan) were obtained. One experienced OCT reader (CB) evaluated all OCT images and excluded scans with poor quality or retinal disease as defined in the OSCAR-IB criteria [24]. The macula was scanned in the horizontal direction in an area of 6 × 6 mm (20 degrees) with 49 b-scans; each b-scan was the average of 15 scans. Macular scans were segmented by one analyst (SK) masked to clinical information using the validated Iowa Reference Algorithm version 3.8.0, which enables calculation of the thickness of ten individual retinal layers for each of the nine regions of the Early Treatment of Diabetic Retinopathy Study (ETDRS) grid (Fig. 1) [31]. Mean thickness of the RNFL and GCL were calculated for three regions: the total EDTRS grid-surface, the pericentral ring (region 2–5) and the peripheral ring (region 6–9) (Fig. 1). The optic nerve head was scanned with a 3.5 mm circle centered on the optic disc, containing 768 × 496 voxels. Peripapillary RNFL (pRNFL) thickness was measured automatically by Heidelberg’s built-in segmentation algorithm (version 1.910.0); both the total peripapillary ring and each of the four quadrants (temporal, superior, nasal and inferior) were used for analysis (Fig. 1). We allowed for inclusion of one eye if the other eye was not eligible for inclusion. If both eyes were eligible, the mean layer thickness of both eyes was used for analysis.

### Statistical analysis

IBM SPSS statistics version 24 (IBM Inc.) was used for all statistical analyses. Data for men and women were analyzed separately. Normality was assessed with visual inspection and using the Shapiro–Wilk test [32]. First, we assessed if there were differences in retinal layer thickness between patients and controls with unpaired Student’s t tests. All data were normally distributed, except for the GCL-pericentral ring data of the male patient subgroup (slightly skewed to the left). As it was a minor deviation from the normal distribution and to increase comparability with the other subgroups, we decided to analyze the data as if it was normally distributed. Indeed, confirmatory non-parametric testing (Mann–Whitney *U *test) showed very similar results for this subgroup. Second, we analyzed differences in retinal layer thickness between three groups (symptomatic patients, asymptomatic patients and controls) with ANOVA (normally distributed data). In case of a significant difference between the groups, post hoc testing was performed with Tukey correction for multiple comparisons. Effect sizes of the differences between groups were quantified by reporting Cohen’s d, which was calculated as the difference between means divided by the pooled standard deviation. A Cohen’s d of 0.2 was considered a small effect, 0.5 a medium effect and 0.8 a large effect [33, 34]. Finally, we correlated clinical outcome measures of severity of myelopathy with the OCT measurements that were able to detect significant between-group differences using Pearson’s correlation (normally distributed continuous data) or Spearman’s rank-order correlation (non-normally distributed continuous data and ordinal data) with a Bonferroni correction for multiple comparisons. To assess the effect of age on retinal layer thickness, we determined correlations between age and retinal layer thickness in the control group. In addition, we performed multiple regression analyses with retinal layer thickness as dependent variable and either age and clinically relevant groups (controls, asymptomatic and symptomatic patients) or age and severity of myelopathy (clinical outcome measures) as independent variables.

For all statistical tests, a significance level of *α* = 0.05 (two-sided) was chosen. Significance levels after correction for multiple comparisons were reported separately.

## Results

Of 148 subjects screened, 132 were included: 62 patients (29 men and 33 women) and 70 controls (33 men and 37 women). For 8 of these 132 subjects, only 1 of both eyes was eligible for inclusion. Online Resource Supplementary File 1 shows details on the number of subjects/eyes excluded and reasons for exclusion. Median age was similar for patients and controls for both men (41.0 versus 41.0, *p* = 0.83) and women (53.0 versus 48.0, *p* = 0.17).

Results of the neurological assessments in this cohort are described in more detail elsewhere [14, 25]. In short, 20/29 men (69%) had both symptoms and signs of myelopathy and were, therefore, classified as symptomatic. The median EDSS was 3.5 (range 0–7.0) and the median SSPROM was 85.5 (range 65–100), indicating moderate disability; the median time on the timed up-and-go was 6.7 s (range 2.6–16.6). Of the 33 women, 16 (48.5%) were symptomatic. Their median EDSS was 3.5 (range 0–6.0), median SSPROM 89.0 (range 71.0–100) and the median time on the timed up-and-go was 4.9 s (range 3.73–21.05).

First, we compared retinal layer thickness between patients and controls (Table 1). In men, the RNFL was significantly thinner in patients compared to controls for both the total grid surface, inner and outer ring (*p* ≤ 0.04, effect sizes between 0.53–0.67), but GCL was not. In addition, both the temporal quadrant (*p* = 0.04, effect size 0.54) and superior quadrant (*p* < 0.05, effect size 0.50) of the pRNFL were thinner in male patients compared to controls. In women, the superior (*p* < 0.001, effect size 0.92) and temporal quadrant (*p* = 0.005, effect size 0.73) of the pRNFL were significantly thinner in patients compared to controls, while the RNFL and GCL did not differ between groups.

**Table 1:** Differences in retinal layer thickness between patients and controls

Men	Retinal layer	Region	Patient (*n* = 29)	Control (*n* = 33)	Mean difference (95%CI)	*p* value	Effect size (Cohen’s *d*)
	RNFL	Total grid surface^a^	34.16 (3.98)	36.68 (3.67)	2.52 (0.58–4.47)	0.01	0.66
	(macula), µm	Pericentral ring	27.32 (1.96)	28.45 (2.26)	1.13 (0.05–2.22)	0.04	0.53
		Peripheral ring	36.54 (4.69)	39.52 (4.23)	2.98 (0.72–5.25)	0.01	0.67
	GCL	Total grid surface^a^	35.97 (4.80)	36.69 (4.17)	0.73 (− 1.55–3.00)	0.53	0.16
	(macula), µm	Pericentral ring	53.74 (9.55)	54.85 (8.79)	1.11 (− 3.55–5.77)	0.64	0.12
		Peripheral ring	31.95 (4.14)	32.59 (3.23)	0.64 (-1.23–2.51)	0.40	0.17
	pRNFL	Total^b^	87.36 (12.50)	91.31 (9.27)	3.94 (− 1.65–9.54)	0.16	0.36
	(optic nerve), µm	Superior	106.41 (16.09)	113.91 (13.62)	7.49 (-0.12–15.11)	0.05	0.50
		Nasal	68.69 (15.77)	66.52 (10.37)	− 2.17 (− 8.95–4.60)	0.64	0.16
		Inferior	111.67 (16.59)	114.80 (16.00)	3.12 (− 5.2–11.47)	0.46	0.19
		Temporal	62.67 (14.84)	70.00 (12.37)	7.33 (0.35–14.31)	0.04	0.54

Women	Retinal layer	Region	Patient (*n* = 33)	Control (*n* = 37)	Mean difference (95%CI)	*p* value	Effect size (Cohen’s *d*)
	RNFL	Total grid surface^a^	36.27 (4.15)	36.81 (2.65)	− 0.54 (− 0.82–1.11)	0.52	0.16
	(macula), µm	Pericentral ring	27.53 (2.25)	27.44 (1.59)	0.09 (− 0.83–1.01)	0.19	0.05
		Peripheral ring	39.24 (4.84)	39.98 (3.15)	− 0.74 (− 2.67–1.19)	0.46	0.18
	GCL	Total grid surface^a^	35.78 (3.68)	36.99 (4.07)	− 1.21 (− 3.07–0.65)	0.20	0.31
	(macula), µm	Pericentral ring	52.48 (6.06)	54.05 (6.17)	− 1.57 (− 4.50–1.35)	0.29	0.26
		Peripheral ring	32.12 (3.43)	33.27 (3.80)	− 1.15 (− 2.88–0.59)	0.19	0.32
	pRNFL	Total^b^	90.37 (9.47)	95.04 (8.16)	− 4.67 (− 8.97–0.36)	0.03	0.53
	(optic nerve), µm	Superior	105.68 (12.66)	118.15 (14.28)	− 12.48 (− 19.14–5.82)	< 0.001	0.92
		Nasal	71.63 (12.81)	68.77 (10.14)	2.86 (− 2.73–8.46)	0.31	0.25
		Inferior	119.13 (17.34)	120.96 (15.01)	− 1.82 (− 9.72–6.07)	0.65	0.11
		Temporal	65.05 (8.86)	72.28 (10.89)	− 7.23 (− 12.16–2.31)	0.005	0.73

Values are summarized as mean (standard deviation). Unpaired *t *test was used to compare groups

*GCL* ganglion cell layer, *pRNFL* peripapillary retinal nerve fiber layer, *S* symptomatic patients, *RNFL* retinal nerve fiber layer

^a^Mean of the total ETDRS grid surface, followed by the pericentral (inner) and peripheral (outer) rings

^b^Mean of the total peripapillary ring followed and each of the four quadrants

Second, we compared the RNFL and pRNFL thickness between controls, asymptomatic patients and symptomatic patients (Table 2). In men, statistically significant overall between-group differences were detected for the RNFL (total grid surface and peripheral ring, *p* ≤ 0.002) and the pRNFL (superior and temporal quadrant, *p* ≤ 0.02). Post hoc testing showed significant differences between symptomatic patients and controls (effect sizes between 0.76–1.02), between symptomatic and asymptomatic patients (effect sizes between 0.96–1.13), but not between asymptomatic patients and controls. In women, only the superior and temporal quadrant of the pRNFL showed significant between-group differences (*p* ≤ 0.02). Post hoc testing for the superior quadrant showed differences between symptomatic patients and controls (effect size 1.13) and symptomatic and asymptomatic patients (effect size 0.77). In contrast, for the temporal quadrant of the pRNFL, a significant difference was detected between asymptomatic patients and controls (effect size 1.01).

**Table 2:** Differences in retinal nerve fiber layer thickness between controls, asymptomatic patients and symptomatic patients

Men	Retinal layer	Region	Control (*n* = 33)	Asymptomatic (*n* = 9)	Symptomatic (*n* = 20)	*p* value	Cohen’s *d* effect size		
							C vs A	C vs S	A vs S
	RNFL, µm	Total grid surface^a^	36.68 (3.67)	36.93 (2.99)	32.91 (3.78)	**0.001**	0.07	**1.02**	**1.13**
		Pericentral ring	28.45 (2.26)	27.77 (1.46)	27.12 (2.16)	0.09	0.32	0.60	0.33
		Peripheral ring	39.52 (4.23)	39.97 (3.57)	35.99 (4.35)	**0.002**	0.11	**0.83**	**0.96**
	pRNFL, µm	Total^b^	91.30 (9.26)	93.13 (8.51)	84.77 (13.30)	0.06	0.20	0.60	0.69
		Superior	113.91 (13.62)	114.56 (12.50)	102.75 (16.44)	**0.02**	0.05	**0.76**	0.77
		Nasal	66.52 (10.37)	67.94 (10.54)	69.02 (13.14)	0.80	0.14	0.22	0.09
		Inferior	114.80 (16.00)	117.89 (10.55)	108.88 (18.22)	0.29	0.21	0.35	0.55
		Temporal	70.00 (13.99)	72.11 (15.31)	58.42 (12.83)	**0.005**	0.14	**0.86**	**1.01**

Women	Retinal layer	Region	Control (*n* = 37)	Asymptomatic (*n* = 10)	Symptomatic (*n* = 23)	*P* value	Cohen’s *d* effect size		
							C vs A	C vs S	A vs S
	RNFL, µm	Total grid surface^a^	36.81 (3.54)	35.73 (3.54)	36.51 (4.44)	0.67	0.31	0.08	0.19
		Pericentral ring	27.44 (1.92)	27.13 (2.58)	27.71 (2.13)	0.72	0.15	0.14	0.26
		Peripheral ring	39.98 (3.15)	38.66 (3.90)	39.49 (5.25)	0.61	0.37	0.11	0.18
	pRNFL, µm	Total^b^	95.04 (8.16)	92.74 (8.62)	89.19 (9.87)	0.06	0.28	0.66	0.37
		Superior	118.16 (14.28)	111.88 (9.82)	102.58 (12.98)	** < 0.001**	0.46	**1.13**	**0.77**
		Nasal	68.77 (10.14)	73.80 (15.88)	70.55 (11.29)	0.60	0.44	0.17	0.26
		Inferior	120.96 (15.01)	122.53 (13.19)	117.44 (19.17)	0.65	0.11	0.21	0.29
		Temporal	72.28 (10.89)	62.75 (7.60)	66.20 (9.39)	**0.01**	**0.93**	0.59	0.39

Values are summarized as mean (standard deviation). ANOVA with Tukey correction for post hoc comparisons was used to compare groups. Effect sizes for post hoc comparisons are displayed and bold if statistically significant

*A* asymptomatic patients, *C* controls, *GCL* ganglion cell layer, *pRNFL* peripapillary retinal nerve fiber layer, *S* symptomatic patients, *RNFL* retinal nerve fiber layer

^a^Mean of the total ETDRS grid surface, followed by the pericentral (inner) and peripheral (outer) rings

^b^Mean of the total peripapillary ring followed and each of the four quadrants

**Fig. 2:**
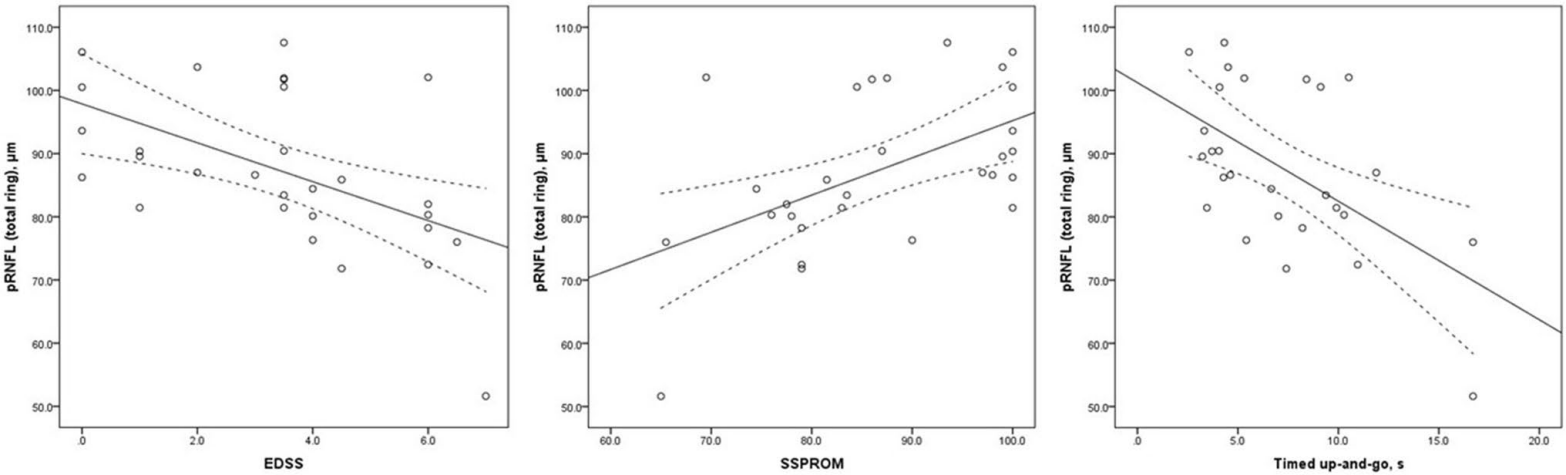
Scatter plots of the relationship between clinical parameters of severity of myelopathy (EDSS, SSPROM and Timed up-and-go) and peripapillary retinal nerve fiber layer (pRNFL) thickness. The continuous lines represent simple linear regression lines and the dotted lines the 95% confidence interval

Finally, we correlated retinal layer thickness to the severity of myelopathy as assessed with the clinical outcome measures (Table 3, Fig. 2). Only the retinal layers that showed significant between-group differences were included in these correlations. In men, there were moderately strong correlations between all three clinical outcome measures (EDSS, SSPROM and timed up-and-go) and both the RNFL (correlation coefficients between 0.43 and 0.48) and the pRNFL (correlation coefficients between 0.29 and 0.55). In women, there were no statistically significant correlations between severity of myelopathy and retinal layer thickness (Online Resource Supplementary File 1), except for the EDSS and the superior quadrant of the pRNFL (correlation coefficient 0.46, *p* = 0.01).

**Table 3:** Correlations between severity of myelopathy and retinal nerve fiber layer thickness in men with ALD

	RNFL (total grid)	RNFL (peripheral ring)	pRNFL (total)	pRNFL (superior)	pRNFL (temporal)
EDSS (*n* = 29)					
Spearman’s rho	− 0.47	− 0.48	− 0.59	− 0.50	− 0.39
*p*-value	0.01	0.008	0.001	0.006	0.04
SSPROM (*n* = 29)					
Spearman’s rho	0.43	0.45	0.54	0.55	0.32
*p*-value	0.02	0.02	0.003	0.002	0.09
Timed up-and-go (n = 27)					
Spearman’s rho	− 0.45	− 0.47	− 0.48	− 0.51	− 0.29
*p*-value	0.02	0.01	0.01	0.007	0.15

All correlations were calculated with Spearman’s rank order correlation test. After Bonferroni correction for multiple comparisons, correlations were considered significant if *p* < 0.025

*EDSS* Expanded Disability Status Score, *RNFL* retinal nerve fiber layer, *pRNFL* peripapillary retinal nerve fiber layer, *SSPROM* Severity Scoring system for Progressive Myelopathy

There were no significant correlations between age and retinal layer thickness in the male or female control group. Regression analysis with both age, sex and group (controls, asymptomatic and symptomatic patients) as independent variables showed a significant effect of group (but not age or sex) on the RNFL (*n* = 132, *B* = − 0.962, *p* = 0.008) and pRNFL temporal quadrant (*B* = − 4.46, *p* = < 0.001) and of both group (*B* = − 5.73, *p* = < 0.001) and age (*B* = 0.260, *p* = 0.004) on the pRNFL superior quadrant. Regression analysis with both age and clinical outcome measures as independent variables was not possible due to a high correlation (collinearity) between age and severity of myelopathy.

## Discussion

In this cross-sectional study, we show that the myelopathy of ALD is associated with thinning of the retinal nerve fiber layer on OCT. While axonal degeneration with thinning of the spinal cord has been demonstrated in ALD patients compared to controls [6], our results show that this axonal degeneration is also measurable in the retina. Moreover, the retinal neurodegeneration correlates with clinical outcome measures of myelopathy. To date, retinal neurodegeneration has been described in a number of neurological diseases [18, 19, 35, 36], but has never been studied in ALD. Demonstrating retinal neurodegeneration in ALD patients and correlating it to clinical measures of severity of myelopathy are important first steps in the validation of OCT as a surrogate outcome measure for myelopathy in ALD.

Although neuroretinal layer thinning was also present in female ALD patients, correlations with clinical outcome measures were less pronounced than in men. This could be explained by the milder disease course of myelopathy in women, who are affected at a higher age and with slower progression than men [2, 8]. In the male subgroup, the absolute differences in retinal layer thickness were largest for the pRNFL (temporal and superior quadrant), while statistical significance was stronger for the RNFL (Tables 1, 2). This is likely due to the larger standard deviations of the pRNFL measurements compared to the RNFL measurements. Although this shows that there is substantial spread of pRNFL thickness between subjects, it is known that the reproducibility of pRNFL measurements within the same subject over time is excellent [37–39]. Therefore, pRNFL measurements can be valuable for measuring disease progression in individual patients over time. The correlations between retinal layer thickness and clinical measures support this hypothesis, as the pRNFL showed the strongest correlation with the clinical outcome measures in men (Table 3).

While the RNFL was thinner in ALD patients compared to controls, the GCL was not. The RNFL consists of the axons of the neurons that relay the information from the retina to the geniculate nucleus in the thalamus; the GCL contains the cell bodies of these neurons. If the same pathological process occurs both in the spinal cord and neuroretina of ALD patients, one would expect that the retrograde axonal degeneration would eventually lead to degeneration of the cell bodies and hence atrophy of the GCL. This could, however, only be a feature of the advanced stages of the disease. Indeed, a subanalysis of GCL thickness in patients with more severe myelopathy (EDSS ≥ 4.5, *n* = 9) did show a trend towards a thinner GCL compared to controls (mean difference 3.4 µm, *p* = 0.054).

Our study has some limitations. First, the possible effect of age on the outcomes needs to be addressed. Both the pRNFL and GCL are described to decrease with age, while this appears to be less pronounced for the RNFL [40, 41]. As patients and controls in our cohort were well age matched, age is unlikely to be a factor for these between-group analyses. Also, comparing three groups (controls, asymptomatic and symptomatic patients), the group-effect remained when including age as a variable in the regression analyses. Alternatively, age could have influenced the correlation between severity of myelopathy and retinal layer thickness. As both prevalence and severity of myelopathy are strongly age dependent (there is strong degree of collinearity), statistically correcting for age was not an option as it would largely cancel out the disease effect. However, the correlations between the clinical outcome measures and retinal layer thickness in our cohort are much stronger than those described between age and retinal layer thickness [40]. Therefore, it is very unlikely that our findings are (solely) due to age. Longitudinal analyses comparing progression rates of patients with a control group would definitively solve this issue.

Besides a possible confounding effect of age, external validity could be a concern. While we used conventional exclusion criteria as defined in the OSCAR-IB criteria [24], this led to exclusion of 12/74 patients (16.2%). For OCT to be used as a surrogate outcome measure in (for example) clinical trials, such an exclusion rate could be problematic. Despite these exclusions, our sample size is relatively large, especially considering the rarity of this disease. Our results are strengthened by the use of equally sized, age- and-sex matched control groups. While OCT studies sometimes use reference values from historical control groups, the use of a control group is much less sensitive to systematic differences in analysis [21]. Also, the thorough clinical characterization of myelopathy in our cohort allows for association of OCT measurements with multiple clinical outcome measures (EDSS, SSPROM and timed up-and-go) that have been previously shown to have good clinical validity characteristics in this cohort [14].

In conclusion, in this study, we show that the myelopathy of ALD is associated with neuroretinal thinning on OCT. While clinical neurological assessments are subject to high inter- and intrarater variability, OCT has excellent test–retest reliability. Moreover, it is a fast and safe assessment that can largely be done automatically. Therefore, OCT may be used to monitor disease progression and serve as a surrogate outcome measure for clinical trials in ALD. Next steps in the validation include longitudinal studies, which are currently ongoing in this cohort.

## Electronic supplementary material

Below is the link to the electronic supplementary material.

Summary of exclusions per group with reasons for exclusion 1 (PDF 117 kb)

Correlations between severity of myelopathy and retinal nerve fiber layer thickness in women with ALD 2 (PDF 101 kb)
