# Correction to: Optical coherence tomography shows neuroretinal thinning in myelopathy of adrenoleukodystrophy

**DOI:** 10.1007/s00415-020-09867-4

**Published:** 2020-05-20

**Authors:** Wouter J. C. van Ballegoij, Sander C. Kuijpers, Irene C. Huffnagel, Henry C. Weinstein, Bwee Tien Poll‑The, Marc Engelen, Carlien A. M. Bennebroek, Frank D. Verbraak

**Affiliations:** 1 Department of Paediatric Neurology/Emma Children’s Hospital, Amsterdam UMC, University of Amsterdam, Meibergdreef 9, 1100DD Amsterdam, The Netherlands; 2 Department of Neurology, OLVG Hospital, Amsterdam, The Netherlands; 3 Department of Ophthalmology, Amsterdam UMC, University of Amsterdam, Amsterdam, The Netherlands

## Correction to: Journal of Neurology (2020) 267:679–687 https://doi.org/10.1007/s00415-019-09627-z

The original version of this article unfortunately contained a mistake. The joint first authorship (Wouter J. C. van Ballegoij, Sander C. Kuijpers and Irene C. Huffnagel) was not mentioned in the paper.

The article note should be:

Wouter J. C. van Ballegoij, Sander C. Kuijpers and Irene C. Huffnagel are contributed equally to this work.

