# Supplementary material for: Optical coherence tomography shows neuroretinal thinning in myelopathy of adrenoleukodystrophy
Source: J Neurol. 2019 Nov 12;267(3):679–87. doi: 10.1007/s00415-019-09627-z (PMC7035302; doi:10.1007/s00415-019-09627-z)
Supplement: Supplementary file 1 — Summary of exclusions per group with reasons for exclusion 1 (PDF 117 kb) [file 415_2019_9627_MOESM1_ESM.pdf]

Supplementary File 1 – Journal of Neurology

Optical coherence tomography shows neuroretinal thinning in myelopathy of adrenoleukodystrophy

Wouter J.C. van Ballegoij, Sander C. Kuijpers, Irene C. Huffnagel, Henry C. Weinstein, Bwee Tien Poll-The, Marc Engelen, Carlien A.M. Bennebroek, Frank D. Verbraak

Corresponding author:

W.J.C. van Ballegoij, MD

Department of Paediatric Neurology

Emma Children's Hospital, Amsterdam UMC, Amsterdam

The Netherlands

E-mail: w.j.vanballegoij@amsterdamumc.nl

	Patient		Control	
**Men**	***ODS (n=6)***	**OD or OS (n=0)**	**ODS (n=2)**	**OD or OS (n=2)**
	dementia		insufficient quality OCT	insufficient quality OCT
	insufficient quality OCT		amblyopia, low visual acuity	subretinal density on OCT
	visual acuity > 0.1 Logmar			
	refractive error >6D			
	keratoconus			
	cerebral ALD			
**Women**	**ODS (n=6)**	**OD or OS (n=2)**	**ODS (n=2)**	**OD or OS (n=3)**
	visual acuity > 0.1 Logmar	amblyopia	visual acuity > 0.1 Logmar	insufficient quality OCT
	cataract (n=2)	visual acuity > 0.1 Logmar	insufficient quality OCT	macular pseudohole
	refractive error >6D (n=2)			macular Pucker
	aphasia			

Supplementary Table 1 Number of exclusions per group with reasons for exclusion
Abbreviations: OD, right eye; OS, left eye; ODS, both eyes
