# Supplementary material for: Optical coherence tomography shows neuroretinal thinning in myelopathy of adrenoleukodystrophy
Source: J Neurol. 2019 Nov 12;267(3):679–87. doi: 10.1007/s00415-019-09627-z (PMC7035302; doi:10.1007/s00415-019-09627-z)
Supplement: Supplementary file 2 — Correlations between severity of myelopathy and retinal nerve fiber layer thickness in women with ALD 2 (PDF 101 kb) [file 415_2019_9627_MOESM2_ESM.pdf]

Supplementary File 2 – Journal of Neurology

Optical coherence tomography shows neuroretinal thinning in myelopathy of adrenoleukodystrophy

Wouter J.C. van Ballegoij, Sander C. Kuijpers, Irene C. Huffnagel, Henry C. Weinstein, Bwee Tien Poll-The, Marc Engelen, Carlien A.M. Bennebroek, Frank D. Verbraak

Corresponding author:
W.J.C. van Ballegoij, MD
Department of Paediatric Neurology
Emma Children's Hospital, Amsterdam UMC, Amsterdam
The Netherlands
E-mail: w.j.vanballegoij@amsterdamumc.nl

		RNFL (total grid)	RNFL (peripheral ring)	pRNFL (total)	pRNFL (superior)	pRNFL (temporal)
EDSS (n=33)	Spearman's rho	0.09	0.07	-0.23	-0.46	0.08
	p-value	0.61	0.68	0.23	**0.01**	0.69
SSPROM (n=33)	Spearman's rho	-0.01	0.01	0.18	0.40	-0.15
	p-value	0.96	0.94	0.36	0.03	0.42
Timed up-and-go (n=31)	Spearman's rho	0.20	0.19	-0.28	-0.41	-0.33
	p-value	0.28	0.30	0.13	0.03	0.08

Supplementary Table 2 Correlations between severity of myelopathy and retinal nerve fiber layer thickness in women with ALD

All correlations were calculated with Spearman's rank order correlation test. After Bonferroni correction for multiple comparisons, correlations were considered significant if $p < 0.025$.

Abbreviations: EDSS, Expanded Disability Status Score; RNFL, retinal nerve fiber layer; pRNFL peripapillary retinal nerve fiber layer; SSPROM, Severity Scoring system for Progressive Myelopathy
